# Prevalence of visual impairment among older Chinese population: A systematic review and meta-analysis

**DOI:** 10.7189/jogh.11.08004

**Published:** 2021-05-01

**Authors:** Minjie Zou, Dongwei Guo, Aiming Chen, Charlotte Aimee Young, Yi Li, Danying Zheng, Guangming Jin

**Affiliations:** 1 State Key Laboratory of Ophthalmology, Zhongshan Ophthalmic Center, Sun Yat-sen University, Guangzhou, China; 2 The Fifth Affiliated Hospital of Sun Yat-sen University, Zhuhai, China; 3 Department of Ophthalmology, Third Affiliated Hospital, Nanchang University, Nanchang, Jiangxi Province, China; 4 Department of Applied Biology and Chemical Technology, Hong Kong Polytechnic University, Hongkong, China

## Abstract

**Background:**

To evaluate the prevalence of visual impairment (VI) among elderly Chinese population.

**Methods:**

All population-based studies on VI prevalence among elderly Chinese populations were searched and only studies with clear definitions of diagnosis were selected. Meta-analysis methods were used to estimate the pooled prevalence and its 95% confidence interval (95%CI) of moderate and severe visual impairment (MSVI) and blindness both by presenting visual acuity (PVA) and best corrected visual acuity (BCVA). Subgroup analysis of gender, district, geographical location, age, education level and examined year were also conducted.

**Results:**

72 studies with 465 039 individuals were included and analyzed. Using PVA, the pooled prevalence of MSVI is 10.9% (95% CI = 9.4%-12.6%) and blindness is 2.2% (95% CI = 1.8%-2.8%), while prevalence of MSVI and blindness by BCVA was 5.4% (95% CI = 4.6%-6.2%) and 2.2% (95% CI = 1.9%-2.5%), respectively. Females, rural residents, older age and lower educational level were risk factors for MSVI and blindness.

**Conclusions:**

VI causes a great health burden among Chinese populations, particularly affecting female subjects, subjects dwelling in rural area, older subjects and subjects with lower educational level.

China, with the largest and fastest aging population in the world, faces a significant challenge in managing age-related eye diseases which can ultimately cause visual impairment (VI) [1-4]. According to the Global Burden of Disease Study 2017, the Disease Adjusted Life Years (DALYs) of VI among Chinese populations has an upward trend, rising from 31.52 (95% CI = 20.55, 47.29) billion in 1990 to 55.12 (95% CI = 36.47, 82.49) billion in 2017, which contributes greatly to the disease burden worldwide [5]. Moreover, the increased life expectancy and the population increase indicates that the prevalence of VI is yet to rise, which would result in heavy economic burden to societies and individuals [6-8].

As a contributing member of the global movement Visual 2020, China has been taking significant strides toward reducing the prevalence of VI [9]. Plenty of past epidemiologic studies have reported on VI prevalence [9,10], and there have been several meta-analyses evaluating the VI prevalence among Chinese population in the past decades [2,11,12]. However, in recent years, many new studies have been conducted, which implies that an update concerning the magnitude of VI prevalence is necessary. Also, as many of the studies reporting VI prevalence are written in Chinese, this information is not accessible to researchers worldwide to assess the current situation of VI prevalence among older Chinese populations.

Therefore, we performed this systematic review and meta-analysis to evaluate the magnitude of VI among older Chinese populations (individuals above 50 years old). Time trends and pooled prevalence of VI together with subgroup analyses by demographic characteristics in older Chinese populations will be investigated in this meta-analysis. We aim to produce results that will provide useful information for appropriate preventive strategies to reduce the disease burden caused by VI in China and beyond.

## METHODS

### Literature search strategy

This meta-analysis was conducted according to the Preferred Reporting Items for Systematic Reviews and Meta-Analyses (PRISMA) statement. Publications reporting prevalence of VI among Chinese populations were reviewed and assessed. Two investigators (ZMJ and GDW) searched for literature independently in both English (Embase, PubMed and Web of science) and Chinese (SinoMed, VIP and Chinese National Knowledge Infrastructure) databases from January 1, 1999 until June 16, 2020. The search terms were as follows:

1. ((“Vision Disorders”[Mesh]) OR “Blindness”[Mesh]) OR (“Vision, Low”[Mesh]) OR (“Visually Impaired Persons”[Mesh]) OR blindness OR visual impairment OR low vision OR visual loss OR visually impaired OR visual disability
2. (“Prevalence”[Mesh]) OR (“Epidemiology”[Mesh]) OR (prevalence OR Epidemiology OR incidence)
3. “China”[Mesh] or China or Chinese
4. Combine 1 AND 2 AND 3

### Study selection

Studies were included if they met the following criteria: 1) population-based study; 2) utilized recognized definitions and standardized grading method to diagnose and classify VI; 3) accessible full text in Chinese or English; 4) explicit survey year; 5) age-specific prevalence data. Population-based studies are those which have a clear sampling frame of the community. The most common definition of VI is as follows: normal vision, visual acuity better than 6/12; moderate and severe visual impairment (MSVI), visual acuity better than 3/60 but worse than 6/18; and blindness, visual acuity worse than 3/60. One sub-lesion of VI, severe visual impairment (SVI), is defined as 3/60 to 6/60. Low vision is defined as having blindness or VI [13]. The prevalence by presenting visual acuity (PVA) and best corrected visual acuity (BCVA) were pooled separately.

Titles and abstracts of all initial searched results were screened independently by two investigators (ZMJ and GDW). If there was more than one publication based on the same study, the study with more thorough information was selected.

### Data extraction and quality assessment

Two investigators (ZMJ and GDW) conducted the data extraction independently and any disagreements were resolved by a discussion with a third investigator (JGM). The following information was extracted and tabulated: first author, study setting, sampling method, survey time, sample size, basic demographic data, the prevalence of MSVI, SVI, VI and SVI if available. For the multicenter surveys, we used the pooled prevalence provided by the original study. If the studies did not provide pooled prevalence, the prevalence data of the single location in the study was used.

The quality of all selected articles was evaluated by two investigators (ZMJ and GDW) with a commonly used 8-item assessment tool[14,15] According to the quality evaluation tool, each study was given a score of 0-8. We consider a score of 7-8 as high quality, 4-6 as moderate quality, and 0-3 as low quality. The coding of assessment has been described previously.

### Statistical analysis

The meta-analysis was conducted using the Comprehensive Meta-Analysis software, Version 2 (Biostat Inc., Englewood, New Jersey, USA). The prevalence of MSVI, SVI (if applicable), VI and blindness with 95% confidence intervals (CI) were calculated using random-effects models if considered of high heterogeneity, otherwise the fixed-effects model was applied. Heterogeneity between studies was assessed by I^2^ statistic, with I^2^>50% regarded as high heterogeneity. Age-specific pooled prevalence of MSVI and blindness by 50-59, 60-69, 70-79, 80 and above years old age groups was conducted. To explore the possible sources of heterogeneity associated with gender, district (rural/urban), geographical location (central/eastern/western China), education level (illiterate/primary school and lower/middle school and above) and survey year (1999-2009/2010-2017), subgroup analyses were performed using Q-tests separately. Continuous variables were dichotomized using median splitting method in subgroup analyses. Publication bias was assessed by the Funnel plots, Begg’s tests and Egger’s tests. Significance level was set at *P* < 0.05(two-tailed) [16].

## RESULTS

### Selection and inclusion of the studies

**Figure 1:**
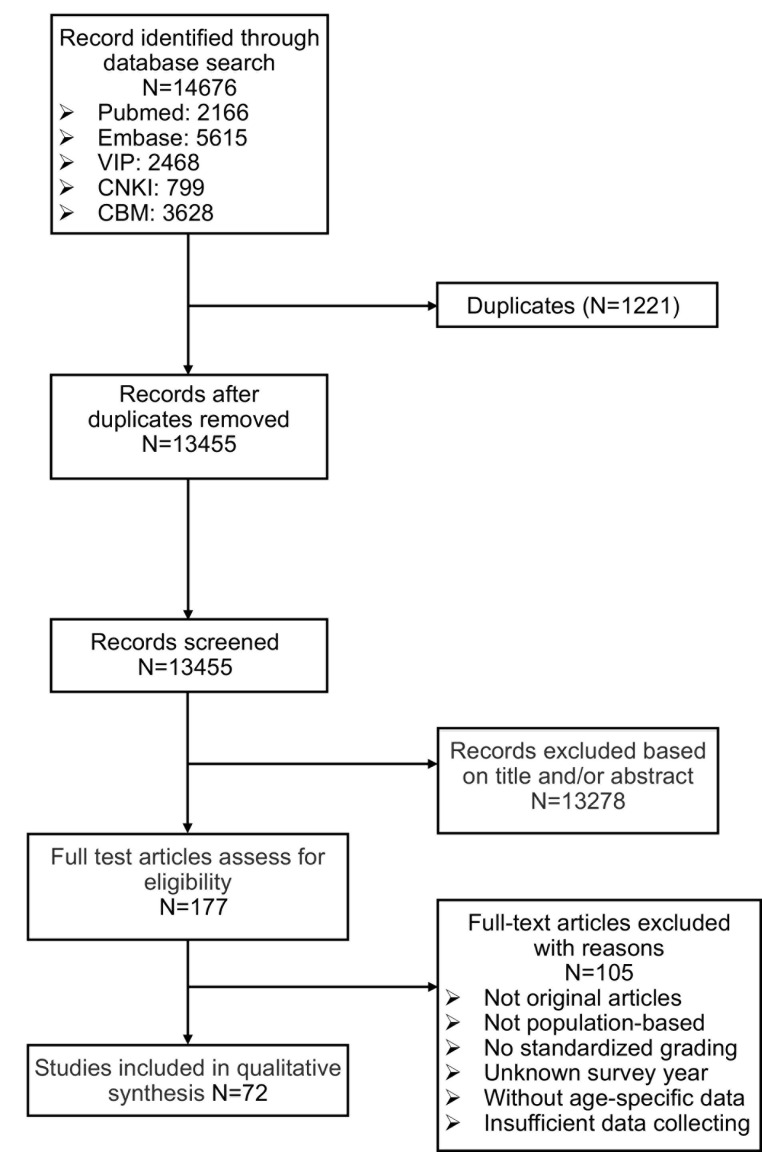
Flowchart of the study selection process.

72 studies with 465 039 individuals out of 14 676 initial records were identified according to the inclusion criteria for analysis (**Figure 1**). There were 90 data sets in total as four studies conducted multi-center surveys with different samples [9,10,17,18]. Basic characteristics of the included studies are given in **Table 1****.** Of these 72 studies, 20 were written in English [9,10,17,19,20,25,27-32,55,56,67-69,75,83,84] and the rest were in Chinese [18,21-24,26,33-54,57-66,70-74,76-83,85-95]. The target population of all selected studies was clearly defined, and all the samples were representative of the general population. 38 studies used BCVA [18,28-64], 9 studies used PVA [19-27] and the rest used both BCVA and PVA [9,10,17,65-95] in the diagnoses of blindness and MSVI. The data of several subgroup analyses (gender, district, geographical location, age, education and examined year) were not available in some included studies; the sum of the individuals may not agree with the total number given by 72 studies.

**Table 1:** Characteristics of included studies

First author	Province	District	Region	Language	Survey year		Age range		N	Measure of visual acuity	Response rate (%)		Definition of older adults	No. of older adults	Assessment*	
Emmy Y. Li [19]	Hainan	E	R	EANG	2010		≥50		6482	PVA	95.3		≥50	6482	6	
CW Pan [20]	Jiangsu	E	R	ENG	2013-2014		≥60		4579	PVA	82.1		≥60	4579	6	
JF Chen [21]	Shanghai	E	R & U	CHN	NA		≥50		5090	PVA	87.8		≥50	5090	5	
H Hu [22]	Yunnan	W	R	CHN	2014		≥50		5592	PVA	93.2		≥50	5592	7	
XJ Zhang [23]	Tianjin	E	R	CHN	2001		≥40		1776	PVA	89.4		≥50	1026	5	
S Liu [24]	Chongqing	W	U	CHN	2005		≥50		5079	PVA	89.4		≥50	5079	6	
L Li [25]	Jiangsu	E	U	ENG	2003		≥60		3040	PVA	90.7		≥60	3040	6	
S Wu [26]	Zhejiang	E	R & U	CHN	2012-2013		≥60		3428	PVA	99.0		≥60	3428	6	
ZJ Li [27]	Heilongjiang	E	R	ENG	2006-2007		≥50		5057	PVA	91.0		≥50	5057	6	
Nancy Chen [28]	Taiwan	E	R	ENG			≥65		2316	BCVA	61.2		≥65	2316	6	
T Li [29]	Shanxi	C	R & U	ENG	2006		0-80		75016	BCVA	85.5		≥50	17473	6	
Y Yao [30]	Jiangsu	E	U	ENG	2010		≥50		6155	BCVA	91.5		≥50	6155	6	
GS Zhang [31]	Inner Mongolia	C	R & U	ENG	2013		≥40		5770	BCVA	80.1		≥50	4928	7	
YG Zhang [32]	Heilongjiang	C	R	ENG	2008-2009		All		10384	BCVA	88.1		≥50	2728	7	
GP Duan [33]	Hunan	C	R	CHN	2008-2009		≥50		4857	BCVA	92.3		≥50	4857	6	
GP Duan [34]	Hunan	C	R	CHN	2008-2009		≥50		4402	BCVA	88.8		≥50	4402	7	
J Fu [35]	Xinjiang	W	R & U	CHN	2009-2010		≥40		8295	BCVA	83.8		≥50	5235	6	
YH Gu [18]	Anhui	C	U	CHN	2008		All		3336	BCVA	91.4		≥50	987	7	
			R						3602		92.1			1386		
XY Huang [36]	Guangdong	E	R	CHN	2012		≥50		4329	BCVA	93.7		≥50	4329	6	
Y Huang [37]	Shanghai	E	R	CHN	2016-2017		≥60		4260	BCVA	70.8		≥60	4260	6	
JP Liu [38]	Yunnan	W	R	CHN	2008		All		2460	BCVA	80.1		≥50	705	8	
XP Liu [39]	Guangdong	E	R & U	CHN	2014-2015		≥50		4539	BCVA	NA		≥50	4539	5	
XY Ma [40]	Shanghai	E	U	CHN	2009		≥65		2299	BCVA	92.0		≥65	2299	6	
M NA Bijiang [41]	Xinjiang	W	U	CHN	2010		≥40		4104	BCVA	81.6		≥50	2853	6	
M Yusup [42]	Xinjiang	W	R	CHN	2009		≥40		4191	BCVA	86.0		≥50	2382	7	
YJ Meng [43]	Tianjin	E	R & U	CHN	2014		≥60		5520	BCVA	94.0		≥60	5520	6	
JZ Pei [44]	Shaanxi	W	R	CHN	2010-2012		All		4394	BCVA	85.0		≥50	1912	7	
H Qi [45]	Shanghai	E	U	CHN	2009		≥60		4785	BCVA	91.3		≥60	4785	6	
LF Qiao [46]	Sichuan	W	R	CHN	2011	≥50		2817		BCVA	98.8	≥50		2817	6
FR Shen [47]	Shanxi	C	R	CHN	NA	≥50		6769		BCVA	96.7	≥50		6769	6
B Shen [48]	Shanghai	E	U	CHN	2010	≥60		6302		BCVA	91.8	≥60		6302	6
W Sun [49]	Jiangsu	E	U	CHN	2010	≥50		6150		BCVA	91.5	≥50		6150	6
GM Wang [50]	Shandong	E	R	CHN	2008	≥50		4916		BCVA	98.2	≥50		4916	6
XL Wu [51]	Zhejiang	E	U	CHN	2015-2016	≥50		5448		BCVA	89.9	≥50		5448	6
LJ Tang [52]	Chongqing	W	U	CHN	2009-2011	≥55		2600		BCVA	NA	≥55		2600	6
SS Zhang [53]	Jiangsu	E	U	CHN	2013	≥60		5564		BCVA	94.9	≥60		5564	6
J Zhang [54]	Shaanxi	W	R	CHN	2007	ALL		8725		BCVA	96.0	≥60		1284	5
XJ Zhang [55]	Guangdong	E	R & U	ENG	2012	≥50		3484		BCVA	94.2	≥50		3484	8
L Xu [56]	Beijing	E	R & U	ENG	2001	≥40		4439		BCVA	83.4	≥50		2987	7
L Chen [57]	Shaanxi	W	R	CHN	2003	≥50		1765		BCVA	80.2	≥50		1765	6
CX Qi [58]	Guangdong	E	U	CHN	2007	≥50		4126		BCVA	91.0	≥50		4126	6
LL Yang [59]	Guangdong	E	U	CHN	2008	≥60		11210		BCVA	88.2	≥60		11210	6
M Wei [60]	Sichuan	W	R & U	CHN	2006	ALL		125641		BCVA	NA	≥50		41441	5
XF Gao [61]	Heilongjiang	E	U	CHN	1999	≥60		7499		BCVA	92.0	≥60		7499	6
X Zhao [62]	Beijing	E	U	CHN	2006	≥50		2410		BCVA	85.1	≥50		2410	5
TY Xie [63]	Xinjiang	W	R	CHN	2005	≥40		2955		BCVA	80.0	≥50		1929	6
TZ Zhang [64]	Xinjiang	W	R	CHN	2001	≥45		1208		BCVA	91.3	≥55		736	5
J Zhou [65]	Jiangsu	E	R	CHN	2008	≥60		1305		PVA&BCVA	93.8	≥60		1305	7
XW Tong [66]	Shanghai	E	U	CHN	2009	≥60		4545		PVA&BCVA	87.4	≥60		4545	6
YT Tang [67]	Zhejiang	E	R & U	ENG	2012-2013	≥45		10234		PVA&BCVA	78.1	≥50		8317	7
J Li [68]	Yunnan	W	R	ENG	2010	≥50		2133		PVA&BCVA	77.8	≥50		2133	8
XF Li [69]	Hebei	E	R	ENG	2010	>7		20298		PVA&BCVA	82.7	≥50		4012	7
M Yang [70]	Jiangsu	E	R	CHN	2010-2011	≥50		5947		PVA&BCVA	96.8	≥50		5947	7
MC Yi [71]	Sichuan	W	U	CHN	2013	≥50		3086		PVA&BCVA	93.7	≥50		3086	7
JY Hu [72]	Jiangsu	E	R	CHN	2006	≥50		653		PVA&BCVA	92.0	≥50		653	6
W Zhou [73]	Shanghai	E	R & U	CHN	2015	≥50		3497		PVA&BCVA	86.3	≥50		3497	5
LH Wang [74]	Shandong	E	R	CHN	2008	≥50		17816		PVA&BCVA	91.0	≥50		17816	8
SS Huang [75]	Guangdong	E	U	ENG	2003	≥50		1399		PVA&BCVA	75.3	≥50		1399	8
BJ Hou [76]	Tibet	W	R	CHN	2000	≥40		3071		PVA&BCVA	97.4	≥50		2060	6
JL Zhao [9]	Multicenter	NA	R	ENG	2014	≥50		51310		PVA&BCVA	90.6	≥50		51310	7
RR Zhu [17]	Jiangsu	E	R	ENG	2010	≥50		5947		PVA&BCVA	96.8	≥50		5947	7
			U					6106			90.8			6106	
W Wang [77]	Shanghai	E	R	CHN	2008-2009	≥60		2150		PVA&BCVA	81.0	≥60		2150	6
CJ Liu [78]	Shanghai	E	U	CHN	2012	≥70		15238		PVA&BCVA	NA	≥70		15238	4
LL Deng [79]	Jiangxi	C	R	CHN	2015	≥50		5119		PVA&BCVA	94.1	≥50		5119	6
JH Zhou [80]	Yunnan	W	R	CHN	2011-2012	≥50		5151		PVA&BCVA	92.4	≥50		5151	5
M Wu [81]	Yunnan	W	R	CHN	2008	≥50		2842		PVA&BCVA	94.7	≥50		2842	5
JL Zhao [10]	Multicenter	NA	R	ENG	2006	≥50		47547		PVA&BCVA	91.5	≥50		47547	8
M Wu [82]	Yunnan	W	R & U	CHN	2006	≥50		2588		PVA&BCVA	93.8	≥50		2588	5
XB Huang [83]	Shanghai	E	U	CHN	2007-2008	≥60		3851		PVA&BCVA	92.7	≥60		3851	6
WL Song [84]	Heilongjiang	E	R	ENG	2007	≥40		4956		PVA&BCVA	86.0	≥50		3525	6
XJ Xiong [85]	Chongqing	W	R	CHN	NA	≥50		2122		PVA&BCVA	83.0	≥50		2122	7
YB Liang [86]	Hebei	E	R	ENG	2006-2007	≥30		6830		PVA&BCVA	90.4	≥50		4241	8

E – Eastern China, C – Central China, W – Western China, R – rural area, U – urban area, ENG – English, CHN – Chinese, NA – not applicable

*The assessment of the studies was conducted using the 8-item assessment tool [14,15].

### Quality assessment and publication bias

The 8-item assessment tool was used to evaluate the quality of the included studies, ranging from 4 to 8, with an average assessment score of 6.2. 7 studies were given the highest score [10,38,55,68,74,75,86], and 23 studies were classified as high quality, while the rest were considered moderate quality. The most common problem of the included studies in the meta-analysis was unclear description of non-responders.

After removing each study sequentially for sensitivity analysis, the pooled prevalence of remaining studies did not change significantly compared to the initial results. According to the results of the Begg’s test (MSVI: Z = 1.524, *P* = 0.127; blindness: Z = 1.676, *P* = 0.094), Egger’s tests (MSVI: *t* = 0.547, *P* = 0.588; blindness: *t* = 0.648, *P* = 0.521) and funnel plot (Figure S1 in the **Online Supplementary Document**, Panels A and B), we concluded that there was no publication bias in both the prevalence of blindness and MSVI by PVA. Concerning the prevalence of blindness and MSVI by BCVA, although the Begg’s tests indicated potential bias may exist (MSVI: Z = 2.940, *P* = 0.003; blindness: Z = 2.525, *P* = 0.012), the Egger’s tests (MSVI: *t* = 0.878, *P* = 0.383; blindness: *t* = 1.305, *P* = 0.197) and funnel plot (Figure S1 in the **Online Supplementary Document****,** Panels C and D**)** did not suggest any publication bias.

### Prevalence of blindness and VI among Chinese population by using PVA

The pooled prevalence using PVA is given by **Table 2** and Figure S1 in the **Online Supplementary Document** (Panels E and F), with 250 080 individuals in total. MSVI prevalence reported in original studies varied from 1.8% to 43.7%[9,25], while blindness was 0.3% to 10.6%[21,76]. The pooled prevalence of MSVI was 10.9% (95% CI = 9.4%-12.6%) and blindness was 2.2% (95% CI = 1.8%-2.8%). As for SVI and VI, the pooled rate was 2.7% (95% CI = 1.9%-3.8%) and 13.6% (95% CI = 11.8%-15.6%).

**Table 2:** Pooled prevalence of visual impairment by presenting visual acuity

First author	No. of older adults	Presenting visual acuity			
**MSVI**	**SVI**	**VI**	**Blindness**
Emmy Y. Li [19]	6482	11.8 (11.0-12.6)	1.9 (1.6-2.2)	16.2 (15.3-17.1)	4.4 (3.9-4.9)
CW Pan [20]	4579	6.3 (5.6-7.0)	–	6.8 (6.1-7.5)	0.5 (0.3-0.8)
JF Chen [21]	5090	3.4 (2.9-3.9)	–	3.7 (3.2-4.2)	0.3 (0.2-0.5)
H Hu [22]	5592	14.7 (13.8-15.7)	1.6 (1.3-1.9)	20.1 (19.0-21.1)	5.4 (4.8-6.0)
XJ Zhang [23]	1026	–	–	–	2.0 (1.3-3.0)
S Liu [24]	5079	7.6 (6.9-8.4)	–	12.9 (12.0-13.9)	5.3 (4.8-6.0)
L Li [25]	3040	1.8 (1.4-2.4)	–	3.2 (2.6.-3.9)	1.4 (1.0-1.8)
S Wu [26]	3428	7.9 (7.1-8.9)	2.6 (2.1-3.2)	10.6 (9.6-11.7)	2.7 (2.2-3.3)
ZJ Li [27]	5057	8.3 (7.6-9.1)	–	10.2 (9.4-11.1)	1.9 (1.6-2.3)
J Zhou [65]	1305	18.6 (16.6-20.8)	–	25.8 (23.4-28.2)	7.1 (5.9-8.7)
XW Tong [66]	4545	8.8 (8.2-9.4)	–	9.7 (8.9-10.6)	0.9 (0.6-1.2)
YT Tang [67]	8317	8.8 (8.2-9.4)	–	10.0 (9.3-10.6)	1.2 (1.0-1.5)
J Li [68]	2133	15.2 (13.8-16.8)	–	18.8 (17.2-20.5)	3.6 (2.9-4.5)
XF Li [69]	4012	9.5 (8.6-10.4)	–	10.9 (10.0-11.9)	1.2 (0.9-1.6)
JL Zhao (2014) [9]	51310	10.3 (10.0-10.6)	–	12.0 (11.7-12.2)	1.7 (1.6-1.8)
RR Zhu [17]	5947	23.6 (22.6-24.7)	2.0 (1.7-2.4)	26.0 (24.9-27.1)	2.3 (2.0-2.7)
	6106	5.4 (4.8-6.0)	0.5 (0.3-0.7)	6.3 (5.7-6.9)	0.9 (0.7-1.2)
M Yang [70]	5947	23.6 (22.6-24.7)	2.0 (1.7-2.4)	25.9 (24.8-27.1)	2.3 (2.0-2.7)
MC Yi [71]	3086	12.5 (11.3-13.7)	2.3 (1.8-2.9)	15.3 (14.1-16.6)	2.9 (2.3-3.5)
JY Hu [72]	653	16.5 (13.9-19.6)	–	22.5 (19.5-25.9)	6.0 (4.4-8.1)
W Zhou [73]	3497	14.8 (13.6-16.0)	–	15.9 (14.7-17.1)	1.1 (0.8-1.5)
LH Wang [74]	17816	7.0 (6.6-7.4)	–	8.6 (8.2-9.0)	1.6 (1.4-1.8)
W Wang [77]	2150	21.7 (20.0-23.5)	–	23.7 (22.0-25.6)	2.0 (1.5-2.7)
CJ Liu [78]	15238	14.5 (14.0-15.1)	1.0 (0.7-1.2)	16.2 (15.6-16.8)	1.7 (1.5-1.9)
LL Deng [79]	5119	17.0 (16.0-18.1)	–	19.2 (18.1-20.3)	2.2 (1.8-2.6)
JH Zhou [80]	5151	14.1 (13.2-15.1)	–	17.8 (16.8-18.9)	3.7 (3.2-4.3)
M Wu [81]	2842	–	–	–	2.0 (1.6-2.6)
JL Zhao (2006) [10]	45747	10.8 (10.5-11.1)	–	13.1 (12.8-13.4)	2.3 (2.2-2.4)
SS Huang [75]	1399	10.1 (8.6-11.8)	0.6 (0.3-1.2)	10.7 (9.2-12.4)	0.6 (0.3-1.2)
BJ Hou [76]	2060	7.9 (6.8-9.2)	–	18.5 (16.9-20.3)	10.6 (9.4-12.0)
M Wu [81]	2588	–	–	–	3.7 (3.0-4.5)
XB Huang [83]	3851	26.8 (25.4-28.2)	–	33.9 (32.4-35.4)	7.2 (6.4-8.0)
WL Song [84]	3525	9.8 (8.9-10.8)	–	12.3 (11.3-13.5)	2.6 (2.1-3.2)
XJ Xiong [85]	2122	11.2 (9.9-12.6)	0.9 (0.5-1.3)	15.2 (13.7-16.8)	4.0 (3.3-4.9)
YB Liang [86]	4241	8.5 (7.7-9.4)	–	10.0 (9.1-10.9)	1.5 (1.2-1.9)
**Pooled prevalence**	250080	10.9 (9.4-12.6)	2.7(1.9-3.8)	13.6 (11.8-15.6)	2.2 (1.8-2.8)

MSVI – moderate and severe visual impairment, SVI – severe visual impairment, VI – visual impairment

### Prevalence of MSVI and blindness among Chinese population by using BCVA

**Table 3** and Figure S1 in the **Online Supplementary Document** (Panels G and H) shows the reported prevalence of MSVI ranged from 1.3% to 32.0% in terms of BCVA [47,96], and for blindness from 0.4% to 8.7% [56,76]. The pooled prevalence of MSVI and blindness was 5.4% (95% CI = 4.6%-6.2%) and 2.2% (95% CI = 1.9%-2.5%), respectively. Meanwhile, the pooled prevalence of SVI was 1.4% (95% CI = 1.0%-1.9%) and VI prevalence was 7.8% (95% CI = 6.9%-8.9%).

**Table 3:** Pooled prevalence of visual impairment by best corrected visual acuity

First author	No. of older adults	Best corrected visual acuity			
**MSVI**	**SVI**	**VI**	**Blindness**
Nancy Chen [28]	2316	4.1 (3.3-4.9)	–	4.9 (4.1-5.8)	0.8 (0.5-1.3)
T Li [29]	17473	1.4 (1.2-1.6)	–	2.0 (1.8-2.2)	0.6 (0.5-0.8)
Y Yao [30]	6155	3.5 (3.1-4.0)	–	6.8 (6.1-7.4)	3.3 (2.9-3.7)
GS Zhang [31]	4928	13.2 (12.3-14.2)	–	19.8 (18.7-20.9)	6.6 (5.9-7.3)
YG Zhang [32]	2728	4.6 (3.9-5.5)	–	6.8 (5.9-7.8)	2.2 (1.7-2.8)
GP Duan [33]	4857	4.5 (4.0-5.2)	–	6.5 (5.8-7.2)	1.9 (1.6-2.3)
GP Duan [34]	4402	4.9 (4.3-5.6)	–	7.2 (6.5-8.0)	2.3 (1.9-2.7)
J Fu [35]	5235	7.2 (6.5-8.0)	–	10.8 (10.0-11.7)	3.6 (3.1-4.1)
YH Gu [18]	987	2.5 (1.7-3.6)	–	7.8 (6.3-9.6)	5.3 (4.1-6.9)
	1386	2.0 (1.4-2.9)	–	4.9 (3.9-6.2)	2.9 (2.1-3.9)
XY Huang [36]	4329	6.8 (6.1-7.6)	–	9.0 (8.2-9.9)	2.2 (1.8-2.7)
Y Huang [37]	4260	7.7 (7.0-8.6)	–	10.5 (9.6-11.5)	2.8 (2.3-3.3)
JP Liu [38]	705	5.3 (3.8-7.2)	–	9.4 (7.4-11.7)	4.1 (2.9-5.9)
XP Liu [39]	4539	7.0 (6.3-7.7)	–	9.1 (8.3-10.0)	2.2 (1.8-2.6)
XY Ma [40]	2299	7.2 (6.2-8.4)	–	10.0 (8.8-11.3)	2.8 (2.2-3.5)
M NA Bijiang [41]	2853	4.7 (4.0-5.5)	–	6.8 (6.0-7.8)	2.1 (1.7-2.7)
Mehriban Yusup [42]	2382	9.0 (7.9-10.2)	–	14.8 (13.4-16.3)	5.8 (5.0-6.9)
YJ Meng [43]	5520	5.3 (4.7-5.9)	–	10.1 (9.3-10.9)	4.8 (4.3-5.4)
JZ Pei [44]	1912	8.1 (7.0-9.4)	–	9.6 (8.3-11.0)	1.5 (1.0-2.1)
H Qi [45]	4785	13.5 (12.6-14.5)	–	15.9 (14.9-16.9)	2.3 (1.9-2.8)
LF Qiao [46]	2817	15.1 (13.8-16.5)	2.5 (1.9-3.1)	19.1 (17.7-20.6)	3.9 (3.3-4.7)
FR Shen [47]	6769	1.3 (1.1-1.6)	–	2.5 (2.1-2.9)	1.2 (0.9-1.5)
B Shen [48]	6302	9.3 (8.6-10.0)	–	10.7 (10.0-11.5)	1.5 (1.2-1.8)
W Sun [49]	6150	3.5 (3.0-4.0)	–	6.8 (6.1-7.4)	3.3 (2.9-3.7)
GM Wang [50]	4916	5.3 (4.7-6.0)	–	7.7 (7.0-8.4)	2.4 (2.0-2.8)
XL Wu [51]	5448	3.3 (2.9-3.9)	–	4.3 (3.8-3.9)	1.0 (0.7-1.2)
LJ Tang [52]	2600	17.7 (16.3-19.2)	–	21.7 (20.2-23.4)	4.0 (3.3-4.9)
SS Zhang [53]	5564	12.7 (11.9-13.6)	–	13.6 (12.7-14.5)	0.9 (0.7-1.2)
J Zhang [54]	1284	–	–	–	5.1 (4.1-6.5)
XJ Zhang [55]	3484	8.0 (7.2-9.0)	1.1 (0.8-1.5)	10.5 (9.5-11.5)	2.4 (2.0-3.0)
L Xu [56]	2987	1.5 (1.1-2.0)	–	1.9 (1.5-2.5)	0.4 (0.2-0.7)
L Chen [57]	1765	3.5 (2.7-4.5)	–	6.2 (5.2-7.4)	2.7 (2.0-3.6)
CX Qi [58]	4126	8.4 (7.6-9.3)	–	14.4 (13.3-15.5)	6.0 (5.3-6.7)
LL Yang [59]	11210	8.3 (7.8-8.8)	–	12.7 (12.1-13.4)	4.5 (4.1-4.8)
M Wei [60]	41441	1.4 (1.3-1.5)	–	3.8 (3.6-4.0)	2.4 (2.2-2.5)
XF Gao [61]	7499	1.3 (1.1-1.6)	–	2.0 (1.7-2.4)	0.7 (0.5-0.9)
X Zhao [62]	2410	5.9 (5.1-6.9)	–	9.3 (8.2-10.6)	3.4 (2.7-4.2)
TY Xie [63]	2955	10.1 (8.8-11.5)	–	16.9 (15.2-18.6)	6.7 (5.7-7.9)
TZ Zhang [64]	1208	8.4 (6.6-10.7)	–	10.6 (8.6-13.0)	2.2 (1.3-3.5)
J Zhou [65]	1305	9.4 (7.9-11.1)	–	13.4 (11.7-15.4)	4.1 (3.1-5.3)
XW Tong [66]	4545	3.2 (2.7-3.7)	–	3.9 (3.3-4.5)	0.7 (0.5-0.9)
YT Tang [67]	10234	5.9 (5.4-6.4)	–	7.0 (6.5-7.6)	1.1 (0.9-1.4)
J Li [68]	2133	7.8 (6.7-9.0)	–	10.6 (9.4-12.0)	2.9 (2.2-3.7)
XF Li [69]	20298	5.7 (5.1-6.5)	–	6.6 (5.9-7.4)	0.9 (0.6-1.2)
JL Zhao (2014) [9]	51310	4.5 (4.3-4.6)	–	5.9 (5.7-6.1)	1.4 (1.3-1.5)
RR Zhu [17]	5947	6.4 (5.8-7.1)	0.7 (0.6-1.0)	8.1 (7.4-8.8)	1.7 (1.4-2.0)
	6106	2.4 (2.1-2.8)	0.2 (0.1-0.4)	3.2 (2.7-3.6)	0.8 (0.6-1.0)
M Yang [70]	5947	6.4 (5.8-7.1)	0.7 (0.6-1.0)	8.0 (7.3-8.7)	1.6 (1.3-1.9)
MC Yi [71]	3086	6.5 (5.7-7.4)	1.0 (0.7-1.4)	8.8 (7.9-9.9)	2.3 (1.8-2.9)
JY Hu [72]	653	4.3 (3.0-6.1)	–	7.5 (5.7-9.8)	3.2 (2.1-4.9)
W Zhou [73]	3497	4.0 (3.4-4.7)	–	4.9 (4.2-5.6)	0.7 (0.5-1.1)
LH Wang [74]	17816	3.7 (3.4-4.7)	–	5.0 (4.7-5.3)	1.3 (1.2-1.5)
W Wang [77]	2150	8.2 (7.1-9.4)	–	9.8 (8.6-11.1)	1.6 (1.2-2.3)
CJ Liu [78]	15238	3.5 (3.3-3.8)	–	5.0 (4.6-5.3)	1.4 (1.2-1.6)
LL Deng [79]	5119	7.2 (6.5-7.9)	–	8.6 (7.9-9.4)	1.5 (1.2-1.8)
JH Zhou [80]	5151	7.7 (7.0-8.5)	–	13.1 (12.2-14.0)	5.3 (4.8-6.0)
M Wu [81]	2842	–	–	–	2.3 (1.8-2.9)
JL Zhao (2006) [10]	45747	5.3 (5.1-5.5)	–	7.23 (7.0-7.5)	1.9 (1.8-2.1)
SS Huang [75]	1399	3.1 (2.3-4.1)	0.1 (0-0.5)	3.6 (2.7-4.7)	0.5 (0.2-1.0)
BJ Hou [76]	3071	4.8 (4.0-5.8)	–	13.6 (12.1-15.1)	8.7 (7.6-10.0)
M Wu [82]	2588	–	–	–	3.2 (2.6-3.9)
XB Huang [83]	3851	14.6 (13.5-15.7)	–	17.1 (15.9-18.3)	2.5 (2.0-3.0)
WL Song [84]	4956	6.6 (5.9-7.5)	–	9.1 (8.1-10.0)	2.4 (2.4-2.0)
XJ Xiong [85]	2122	6.2 (5.2-7.3)	0.8 (0.5-1.2)	8.0 (6.9-9.2)	1.8 (1.3-2.5)
YB Liang [86]	6830	2.6 (2.2-3.1)	–	3.8 (3.3-4.4)	1.2 (0.9-1.6)
**Pooled prevalence**	438927	5.4 (4.6-6.2)	1.4 (1.0-1.9)	7.8 (6.9-8.9)	2.2 (1.9-2.5)

MSVI – moderate and severe visual impairment, SVI – severe visual impairment, VI – visual impairment

### Subgroup analyses of the pooled prevalence of VI and blindness

**Table 4** shows the subgroup analyses of MSVI and blindness among Chinese population by PVA and BCVA.

**Table 4:** Subgroup analysis of blindness and visual impairment of Chinese population by presenting visual acuity and best corrected visual acuity

			Moderate and severe visual impairment							Blindness			
**Subgroup**	**N**		**Prevalence and 95% CI (%)**		**Heterogeneity, I^2^ (%)**	**Q-value**		***P*-value**	**N**	**Prevalence and 95% CI (%)**	**Heterogeneity, I^2^ (%)**	**Q-value**	***P*-value**
**Prevalence by present visual acuity**													
**Gender:**													
Female	34		15.6 (12.7-18.9)		99.568	7639.915		<0.001	37	2.2 (1.9-2.7)	96.271	965.294	<0.001
Male	34		12.3 (10.0-15.2)		99.378	5307.023			37	1.7 (1.3-2.1)	96.596	1057.694	
**District:**													
Rural	35		14.9 (12.0-18.4)		99.734	12760.453		<0.001	38	2.2 (1.8-2.7)	97.946	1801.707	<0.001
Urban	6		9.0 (5.4-14.8)		99.584	1202.177			7	2.2 (1.2-4.0)	98.923	557.320	
**Geographical location:**													
Central China	3		19.7 (12.0-30.6)		99.535	430.343		<0.001	3	1.7 (1.3-2.4)	85.850	14.134	<0.001
Eastern China	32		12.3 (9.4-15.9)		99.770	13482.959			33	1.6 (1.3-2.1)	97.447	1253.332	
Western China	13		14.1 (10.6-18.3)		99.480	2308.491			15	3.3 (2.5-4.4)	97.901	666.884	
**Age:**													
50-59	36		5.4 (4.3-6.9)		98.914	3223.944		0.165	38	0.7 (0.5-0.9)	91.569	438.851	<0.001
60-69	43		10.3 (8.1-12.9)		99.318	6156.620			45	1.4 (1.0-1.9)	96.445	1237.792	
70-79	39		25.3 (20.9-30.3)		99.356	5902.461			41	3.5 (2.9-4.1)	94.389	712.873	
80+	39		44.2 (36.3-52.4)		99.286	5319.924			41	8.8 (7.1-11.0)	96.407	1113.256	
**Educational level:**													
Illiterate	16		19.1 (15.9-22.8)		98.598	1070.033		<0.001	16	4.8 (3.6-6.4)	96.551	434.917	<0.001
Primary school and lower	20		9.7 (7.7-12.3)		98.364	1161.541			19	1.7 (1.1-2.5)	94.095	423.386	
Secondary school and above	25		6.9 (4.8-9.7)		97.691	1161.378			26	1.7 (1.1-2.7)	96.310	487.763	
**Examined year:**													
1999-2009	27		9.9 (8.4-11.8)		99.027	2670.885		<0.001	30	2.4 (1.9-3.0)	97.858	1353.906	<0.001
2010-2017	24		16.2 (12.6-20.5)		99.763	9699.090			24	1.6 (1.2-2.1)	97.647	977.293	
**Prevalence by best corrected visual acuity**													
**Gender:**													
Female		48		7.1 (5.9-8.4)	99.029		4840.553	<0.001	49	1.9 (1.6-2.3)	97.391	1839.996	<0.001
Male		48		5.6 (4.7-6.8)	98.637		3447.731		49	1.6 (1.3-1.9)	95.571	1083.737	
**District:**													
Rural		53		6.2 (5.2-7.3)	99.199		6492.752	<0.001	54	1.9 (1.6-2.3)	97.563	2174.966	<0.001
Urban		23		4.6 (3.5-6.2)	99.280		3053.625		23	1.7 (1.3-2.3)	97.807	1002.969	
**Geographical location:**													
Central China		11		4.3 (2.6-7.0)	99.466		1872.103	<0.001	11	2.0 (1.2-3.3)	98.552	690.498	<0.001
Eastern China		45		5.9 (4.9-7.1)	99.312		6459.533		45	1.6 (1.3-1.9)	97.311	1636.031	
Western China		22		7.2 (5.4-9.7)	99.388		3429.291		25	2.9 (2.3-3.6)	97.463	946.153	
**Age:**													
50-59		56		1.8 (1.5-2.2)	95.091		1120.306	<0.001	56	0.6 (0.4-0.8)	94.379	978.466	<0.001
60-69		67		4.3 (3.7-4.9)	96.857		2100.172		68	1.4 (1.1-1.8)	96.959	2203.248	
70-79		56		10.9 (9.0-13.0)	98.793		4556.433		56	2.9 (2.4-3.5)	96.118	1416.784	
80+		56		22.5 (18.1-27.6)	99.086		6020.206		56	7.6 (6.2-9.3)	96.149	1428.325	
**Education:**													
Illiterate		16		9.7 (8.2-11.5)	96.010		375.901	<0.001	16	3.4 (2.5-4.5)	96.217	396.544	<0.001
Primary school and lower		17		4.4 (3.2-5.2)	97.325		598.045		17	1.4 (1.0-2.0)	92.566	215.214	
Secondary school and above		23		3.6 (.4-5.2)	96.319		597.665		23	1.1 (0.7-1.7)	89.597	211.484	
**Examined year:**													
1999-2009		40		4.9 (4.1-6.0)	98.993		3873.349	<0.001	43	2.1 (1.8-2.6)	97.764	1878.643	<0.001
2010-2017		34		7.9 (6.5-9.6)	99.321		4859.186		34	1.8 (1.4-2.2)	97.915	1582.527	

CI – confidence interval

**Figure 2:**
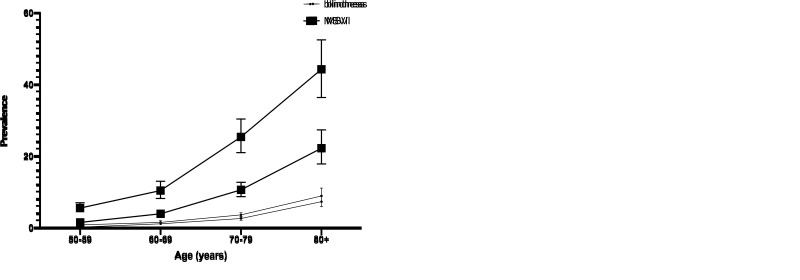
Age-and gender-specific prevalence of MSVI and Blindness. **Panel A.** Age-and gender-specific prevalence of MSVI in term of PVA and BCVA. **Panel B.** Age-and gender-specific prevalence of blindness in term of PVA and BCVA. See also Figure S1 in the **Online Supplementary Document**.

For MSVI, subgroup analyses using PVA for diagnosis presented similar results with that of BCVA. Gender difference is of statistical significance, as the prevalence of females (15.6%, 95% CI = 12.7%-18.9% for PVA; 7.1%, 95% CI = 5.9%-8.4% for BCVA) surpassed those of male (12.3%, 95% CI = 10.0%-15.2% for PVA; 5.6%, 95% CI = 4.7%-6.8% for BCVA). In geographical subgroup analysis, MSVI prevalence among the rural part of China is higher than the urban area (*P* < 0.001). Meanwhile, for the subgroup analysis of age group using PVA for diagnosis, the MSVI prevalence rose from 5.4% (95% CI = 4.3%-6.9%) in the 50-59 age group to 44.2% (36.3%-52.4%) in the 80+ age group, given by **Figure 2**, Panel A. As for BCVA, prevalence of MSVI increased from 1.8% (95% CI = 1.5%-2.2%) to 22.5% (95% CI = 18.1%-27.6%) (*P* < 0.001). In terms of education level, populations that had obtained higher education were at a lower risk of developing MSVI both by PVA and BCVA (*P* < 0.001). As for survey year, studies conducted in early years (1999-2009) had a lower MSVI prevalence than those conducted later (2010-2017) with statistical significance by PVA and BCVA (*P* < 0.001). Studies conducted during 2010 and 2017 had a lower prevalence compared to those conducted between 1999 and 2009 with statistical significance (*P* < 0.001).

In the blindness subgroup analyses, some results were similar to MSVI, as females, rural residents, and lower educational level were risk factors of developing blindness (*P* < 0.001). For example, the blindness prevalence by PVA climbed up from 0.7% (95% CI = 0.5%-0.9%) among 50-59 age group to 8.8% (7.1%-11.0%) among individuals over 80 years old (**Figure 2****,** Panel-B). However, people dwelling in Western China were more likely to develop blindness by PVA (3.3%, 95% CI = 2.5%-4.4%, *P* < 0.001). Unlike MSVI, studies conducted during 2010 and 2017 had a lower prevalence compared to those conducted between 1999 and 2009 with statistical significance (*P* < 0.001).

## DISCUSSION

In our study, 72 studies with 90 data sets conducted in different parts of China were included, and the pooled prevalence of VI, MSVI, SVI and blindness among older Chinese populations (aged 50 years and above) were assessed. Using PVA as the classification index, prevalence of MSVI, SVI, VI and blindness were, 10.9% (95% CI = 9.4%-12.6%), 2.7% (95% CI = 1.9%-3.8%), 13.6% (95% CI: 11.8%-15.6%) and 2.2% (95% CI = 1.8%-2.8%), respectively. As for BCVA, the pooled MSVI, SVI, VI and blindness prevalence were, 5.4% (95% CI = 4.6%-6.2%), 1.4% (95% CI = 1.0%-1.9%), 7.8% (95% CI = 6.9%-8.9%) and 2.2% (95% CI: 1.9%-2.5%), respectively. Generally, the results of this meta-analysis are consistent with prior studies showing that blindness and MSVI occur more frequently among the older Chinese population [2,11,12].

In the subgroup analysis of gender, the pooled prevalence of females was much higher than that of male, in accordance with most of the original investigations. One possible explanation could be the longer life expectancy of females, which accounts for higher risk of developing age-related ocular diseases [1,3]. Additionally, anatomical and hormonal differences may contribute to the gender difference, as females are proved to be at higher risk of developing cataract, one of the common ocular diseases that leads to MSVI and blindness, but the mechanism is yet to be elucidated [97-99]. Also, the social status of females is much lower in some remote areas, leading to lower quality of health care.

Residing in specific geographical area may contribute to the development of blindness and MSVI; in this study dwelling in rural areas or Western China is considered to be an important risk factor. One explanation is the shortage of health care services in less developed and geographically remote places. Lack of health awareness is also a significant factor among rural residents [100]. The pooled MSVI prevalence of Central China seems higher than those of Western and Eastern China by PVA, however, this may be caused by the limited included studies conducted in Central China. Therefore, these results could assist in guiding the development and implementation of health care resources and policies to focus more on rural populations and promoting health awareness.

The significant difference of the prevalence of MSVI and blindness in the 4 age groups should be highlighted. The pooled prevalence rate were 10 times higher in the 80+ age group than the 50-59 age group. As we know, MSVI and blindness are mainly caused by age-related ocular diseases [1,3,4]. Screenings and early diagnosis methods are vital for reducing the prevalence of MSVI and blindness, and will improve the quality of life of older populations.

When comparing groups of education levels, individuals with higher education were less likely to suffer from MSVI and blindness. Education level is considered to be strongly correlated with socioeconomic status, which is correlated with access to quality medical services [101,102]. Higher education may also be linked to a better understanding and awareness of MSVI and blindness, resulting in timely treatment after the appearance of relevant symptoms [103,104]. Educational intervention plays an important role in raising awareness of the severity of MSVI and blindness in the general population.

In our study, the prevalence of MSVI is trending upward while the prevalence of blindness is downward trending when comparing studies surveyed in 1999-2009 with those conducted in 2010-2017. While the cataract surgery rate is trending upward, and the awareness of blindness prevention may help with the reducing of MSVI prevalence [105], the aging population is linked with more individuals at risk of blindness causing ocular diseases, such as age-related macular degeneration and diabetic retinopathy, where treatment in late-stage progression is often of limited efficacy [16,106]. Multiple strategies should be utilized to mitigate these problems, such as better screening strategies and improved education around preventing MSVI and blindness to the general public.

The strength of this meta-analysis lies in the large pooled sample size from a wide geographical distribution. In addition, this is one of the few meta-analyses that includes a significant amount of recently published Chinese studies, allowing for researchers worldwide to assess the current situation of MSVI and blindness among older Chinese populations. Moreover, our study is the first to include both PVA and BCVA. The quality assessment of all included studies with clearly defined evaluation tools should also be highlighted, ensuring the quality of this meta-analysis.

However, several limitations should be considered. Some relevant information in subgroup analysis, such as education level, was not available in all selected articles, which could affect the results to a certain extent. Meanwhile, although the Egger’s tests and funnel plot did not suggest any publication bias, Begg’s tests indicated potential bias may exist in pooled prevalence of blindness and MSVI by BCVA, which may have some impact on the results. Additionally, although we have included both PVA and BCVA as a diagnosis index, using PVA and BCVA in one study is not as common, which may influence the analysis process. The comparison of the accuracy and practicability of PVA and BCVA was not able to be conducted this time, though we will consider this in future studies.

In conclusion, this meta-analysis offers a comprehensive and up-to-date estimate of MSVI and blindness among older Chinese populations, with the subgroups of gender, district, geographical location, education level and survey year analyzed. The results of this meta-analysis indicate that the prevalence of MSVI and blindness remains high and with discrepancy in different subgroups. Further studies are needed to explore improved diagnosis methods and the mechanism of risk factors affecting MSVI and blindness prevalence.

## Additional material

Online Supplementary Document

## References

[1] Fang EF Scheibye-Knudsen M Jahn HJ Li J Ling L Guo H A research agenda for aging in China in the 21st century. Ageing Res Rev. 2015;24:197-205. 10.1016/j.arr.2015.08.003 26304837 PMC5179143

[2] Cheng CY Wang N Wong TY Congdon N He M Wang YX Prevalence and causes of vision loss in East Asia in 2015: magnitude, temporal trends and projections. Br J Ophthalmol. 2020;104:616-22. 10.1136/bjophthalmol-2018-313308 31462416

[3] Peng X China’s demographic history and future challenges. Science. 2011;333:581-7. 10.1126/science.1209396 21798939

[4] Keeffe J Taylor HR Fotis K Pesudovs K Flaxman SR Jonas JB Prevalence and causes of vision loss in Southeast Asia and Oceania: 1990-2010. Br J Ophthalmol. 2014;98:586-91. 10.1136/bjophthalmol-2013-304050 24407561

[5] GBD 2017 DALYs and HALE Collaborators Global, regional, and national disability-adjusted life-years (DALYs) for 359 diseases and injuries and healthy life expectancy (HALE) for 195 countries and territories, 1990-2017: a systematic analysis for the Global Burden of Disease Study 2017. Lancet. 2018;392:1859-922. 10.1016/S0140-6736(18)32335-3 30415748 PMC6252083

[6] Wang W Yan W Muller A Keel S He M Association of Socioeconomics With Prevalence of Visual Impairment and Blindness. JAMA Ophthalmol. 2017;135:1295-302. 10.1001/jamaophthalmol.2017.3449 29049446 PMC6583541

[7] Fenwick EK Ong PG Man RE Cheng CY Sabanayagam C Wong TY Association of Vision Impairment and Major Eye Diseases With Mobility and Independence in a Chinese Population. JAMA Ophthalmol. 2016;134:1087-93. 10.1001/jamaophthalmol.2016.2394 27467140

[8] Bourne RRA Flaxman SR Braithwaite T Cicinelli MV Das A Jonas JB Magnitude, temporal trends, and projections of the global prevalence of blindness and distance and near vision impairment: a systematic review and meta-analysis. Lancet Glob Health. 2017;5:e888-97. 10.1016/S2214-109X(17)30293-0 28779882

[9] Zhao J Xu X Ellwein LB Cai N Guan H He M Prevalence of Vision Impairment in Older Adults in Rural China in 2014 and Comparisons With the 2006 China Nine-Province Survey. Am J Ophthalmol. 2018;185:81-93. 10.1016/j.ajo.2017.10.016 29102607 PMC6029940

[10] Zhao J Ellwein LB Cui H Ge J Guan H Lv J Prevalence of vision impairment in older adults in rural China: the China Nine-Province Survey. Ophthalmology. 2010;117:409-16. 10.1016/j.ophtha.2009.11.023 20079923 PMC6029941

[11] Cheng JW Cheng SW Cai JP Li Y Wei RL The prevalence of visual impairment in older adults in mainland China: a systemaxtic review and meta-analysis. Ophthalmic Res. 2013;49:1-10. 10.1159/000327144 22965304

[12] Song P Wang H Theodoratou E Chan KY Rudan I The national and subnational prevalence of cataract and cataract blindness in China: a systematic review and meta-analysis. J Glob Health. 2018;8:010804. 10.7189/jogh.08.010804 29977532 PMC6005639

[13] Stevens GA White R Flaxman S Price H Jonas J Keeffe J Global prevalence of vision impairment and blindness: magnitude and temporal trends, 1990-2010. Ophthalmology. 2013;120:2377-84. 10.1016/j.ophtha.2013.05.025 23850093

[14] Yang C Zhang L Zhu P Zhu C Guo Q The prevalence of tic disorders for children in China: A systematic review and meta-analysis. Medicine (Baltimore). 2016;95:e4354. 10.1097/MD.0000000000004354 27472724 PMC5265861

[15] Loney PL Chambers LW Bennett KJ Roberts JG Stratford PW Critical appraisal of the health research literature: prevalence or incidence of a health problem. Chronic Dis Can. 1998;19:170-6. 10029513

[16] Jin G Zou M Chen A Zhang Y Young CA Wang SB Prevalence of age-related macular degeneration in Chinese populations worldwide: A systematic review and meta-analysis. Clin Exp Ophthalmol. 2019;47:1019-27. 10.1111/ceo.13580 31268226

[17] Zhu RR Shi J Yang M Guan HJ Prevalences and causes of vision impairment in elderly Chinese: a socioeconomic perspective of a comparative report nested in Jiangsu Eye Study. Int J Ophthalmol. 2016;9:1051-6. 27500116 10.18240/ijo.2016.07.19 PMC4951662

[18] Gu Y Wen Y Liang L Wang L Epidemiological sampling survey on blindness and low vision in two locations of Anhui. [In Chinese]. Journal of Practical Preventing Blind. 2010;5:24-31.

[19] Li EY Liu Y Zhan X Liang YB Zhang X Zheng C Prevalence of blindness and outcomes of cataract surgery in Hainan Province in South China. Ophthalmology. 2013;120:2176-83. 10.1016/j.ophtha.2013.04.003 23714323

[20] Pan CW Qian DJ Sun HP Ma Q Xu Y Song E Visual Impairment among Older Adults in a Rural Community in Eastern China. J Ophthalmol. 2016;2016:9620542. 10.1155/2016/9620542 27777793 PMC5061962

[21] Chen J Xiao Y Zhang Y Analysis of the Vision Condition of People Above 50 years old in Rural-urban Continuum, Shanghai. [In Chinese]. Chinese Primary Health Care. 2016;30:41-6.

[22] Hu H. Epidemiological survey of blindness and low vision among people aged 50 and over in Luxi County of Yunnan Province [In Chinese] [Master Degree]. Kunming Medical University; 2016.

[23] Zhang X Sun H Li Z Zhao L Yuan X Ma Z the investigation of prevalence and causes of blindness among people aged 40 or above in Sangzi, Ji County, Tianjin. [In Chinese]. Zhonghua Yan Ke Za Zhi. 2005;22:749-50.

[24] Liu S Chen L Ouyang L Peng Q The survey of prevalence of blindness in Nan’an District of Chongqing. [In Chinese]. Zhonghua Yan Ke Za Zhi. 2007;43:722-5. 18001571

[25] Li L Guan H Xun P Zhou J Gu H Prevalence and causes of visual impairment among the elderly in Nantong, China. Eye (Lond). 2008;22:1069-75. 10.1038/eye.2008.53 18464798

[26] Wu S Qiu Y Sun X The investigation and risk factors of visual impairment among the elderly in Jiashan County, Zhejiang Province. [In Chinese]. Pract Prev Med. 2015;22:333-4.

[27] Li Z Cui H Liu P Zhang L Yang H Zhang L Prevalence and causes of blindness and visual impairment among the elderly in rural southern Harbin, China. Ophthalmic Epidemiol. 2008;15:334-8. 10.1080/09286580802227386 18850470

[28] Chen N Huang TL Tsai RK Sheu MM Prevalence and causes of visual impairment in elderly Amis aborigines in Eastern Taiwan (the Amis Eye Study). Jpn J Ophthalmol. 2012;56:624-30. 10.1007/s10384-012-0178-8 22961342

[29] Li T Du L Du L Prevalence and Causes of Visual Impairment and Blindness in Shanxi Province, China. Ophthalmic Epidemiol. 2015;22:239-45. 10.3109/09286586.2015.1009119 26218106

[30] Yao Y Shao J Sun W Zhu J Fu DH Guan H Prevalence of blindness and causes of visual impairment among adults aged 50 years or above in southern Jiangsu province of China. Pak J Med Sci. 2013;29:1203-7. 24353720 10.12669/pjms.295.3866 PMC3858935

[31] Zhang G Li Y Teng X Wu Q Gong H Ren F Prevalence and causes of low vision and blindness in Baotou: A cross-sectional study. Medicine (Baltimore). 2016;95:e4905. 10.1097/MD.0000000000004905 27631267 PMC5402610

[32] Zhang Y Wang H Liu J Wang T Cao S Zhou D Prevalence of blindness and low vision: a study in the rural Heilongjiang Province of China. Clin Exp Ophthalmol. 2012;40:484-9. 10.1111/j.1442-9071.2011.02682.x 21902783

[33] Duan G. Epidemiological Survey on Low Vision and Blindness in Persons More than 50 Years Old in Fenghuang County of Hunan Province. [In Chinese]. Journal of Clinical Research. 2011;28:1452-4.

[34] Duan G Wang Y He G Yang J Li A Li J Epidemiological Survey of Low Vision and Blindness in the Population Aged 50 Years and Above in Huayuan County, Hunan Province. [In Chinese]. Pract Prev Med. 2011;18:1423-5.

[35] Fu J Xie T Zhang M Mi N Mai D Chen X Prevalence of low vision and blindness in defined population in rural and urban areas in Xinjiang. [In Chinese]. Chinese Journal of Practical Ophthalmology. 2012;30:756-60.

[36] Huang X Ye Q Liang X Lin R Li L Zeng Z The epidemiological investigation of blindness and low vision among individuals aged 50 or above in Taiping county, Mazhang District, Zhanjiang. [In Chinese]. Journal of Guangdong Medical College. 2014;32:105-7.

[37] Huang Y. Reasons of visual impairment and cataract surgical rate of elder people aged 60 years old or more in Shuxin Town, Chongming District, Shanghai. [In Chinese]. China Medical Herald. 2018;15:101-7.

[38] Liu JP Zhao S Li X Wei R Wang T Hua N Prevalence survey of visual impairment in a multiethnic rural district in the high altitude area of Yunnan Province, China. [In Chinese]. Zhonghua Yan Ke Za Zhi. 2011;47:791-6. 22177123

[39] Liu X Li Y Liu D Luo S Lu Y Liang Y The epidemiological investigation of blindness and visual impairment among individuals aged 50 or above in Foshan. [In Chinese]. Guangdong Yixue. 2016;37:422-5.

[40] Ma X An epidemiological investigation of blindness and vision impairment in older adults of Luwan District, Shanghai. [In Chinese]. Chinese Journal of Disease Control & Prevention. 2012;16:658-60.

[41] Bijiang MN Survey of blindness and low vision among residents in Shuimogou District, Urumqi, China in 2010. [In Chinese]. Zhonghua Yi Xue Za Zhi. 2012;92:743-7. 22781353

[42] Yusup M. Tracely survey of visual damage among Uigur peasants in Kuche county of Xinjiang [In Chinese] [Master Degree]: Xinjiang Medical University; 2010.

[43] Meng Y The prevalence and causes of blindness and visual impairment among individuals aged 60 or above in Tianjin. [In Chinese]. Zhongguo Laonianxue Zazhi. 2016;36:176-8.

[44] Pei J He Y Ren B Jia J Liu H Wan P Investigation of blindness and low vision prevalence in a rural population in Shaanxi Province. [In Chinese]. Recent Advances in Ophthalmology. 2014;34:643-6.

[45] Qi H An epidemiological survey on blindness and low vision among adults aged 60 years or above in Lujiazui blocks, Pudong New Area. [In Chinese]. Chinese Journal of Disease Control & Prevention. 2010;14:748-50.

[46] Qiao L Rapid assessment of avoidable blindness in Mianning county in Sichuan Province. [In Chinese]. Journal of Practical Preventing Blind. 2018;13:125-30.

[47] Shen F Wang C Liang Q Chen C Investigation of Ophthalmopathy in Adults Aged over 50 in Sanhe Town of Jincheng city. [In Chinese]. Chinese Healthcare Innovation. 2010;5:94-6.

[48] Shen B Morbidity survey of blindness and low vision among senior residents in Huaihai communities of Shanghai. [In Chinese]. Chinese Journal of General Practitioners. 2013;12:383-4.

[49] Sun W Causes of blindness and low vision of the people over 50 years old in Binhu Area of Wuxi City. [In Chinese]. Chinese Journal of Ocular Fundus Diseases. 2012;28:602-5.

[50] Wang G Zhou C Bi H Xu L Survey on Prevalence and Causes of Blindness and Low Vision Among Rural Adults Aged 50 Years and above, Tengzhou City, 2008. Preventive Medicine Tribune. 2012;18:574-5.

[51] Wu X Yi Q Wu Y Wang Y Yuan J Guo W Epidemiological investigation of ophthalmopathy among adults aged 50 years or above in Ningbo. [In Chinese]. Chinese Journal of General Practice. 2019;17:491-5.

[52] Tang L Deng R The investigation of ophthalmopathy among individuals aged 55 or above in Chongqing. [In Chinese]. Zhongguo Laonianxue Zazhi. 2012;32:1465-6.

[53] Zhang S Jia S Cao Z Cross-sectional Study on Blindness and Low VIsion Among the Elderly in Xiangcheng District, Suzhou. [In Chinese]. Journal of Occupational Health and Damage. 2014;29:75-7.

[54] Zhang J Epidemiological survey of blindness in Foping of Shaanxi province. [In Chinese]. Int J Ophthalmol. 2008;8:1416-7.

[55] Zhang X Li EY Leung CKS Musch DC Tang X Zheng C Prevalence of visual impairment and outcomes of cataract surgery in Chaonan, South China. PLoS One. 2017;12:e0180769. 10.1371/journal.pone.0180769 28797099 PMC5552304

[56] Xu L Wang Y Li Y Wang Y Cui T Li J Causes of blindness and visual impairment in urban and rural areas in Beijing: the Beijing Eye Study. Ophthalmology. 2006;113:1134.e1-11. 10.1016/j.ophtha.2006.01.035 16647133

[57] Chen L Ren B Yang J He Y Sun N Prevalence of Blindness and Cataract surgery in rural population in Shaanxi Province. [In Chinese]. Chinese Journal of Practical Ophthalmology. 2006;24:648-53.

[58] Qi C Li Q Wang T Dong L Deng J Xia X Epidemiologic survey of blindness and low vision in Luogang country of Guangzhou. [In Chinese]. Chinese Journal of Practical Ophthalmology. 2008;26:1144-6.

[59] Yang L Tu X Hu H Xiao S Epidemiological survey of low vision and blindness of senile persons in Lecong town, Shunde blocks, Guangdong province. [In Chinese]. China Journal of Modern Medicine. 2009;19:2012-4.

[60] Wei M Lei C Chen H Fan Y Analysis of the situation of visual disability of Sichuan Province. [In Chinese]. Int J Ophthalmol. 2007;7:1652-4.

[61] Gao X Duan Z The epidemiological investigation of low vision and blindness among the elderly in some areas of Harbin. [In Chinese]. Harbin Medical Journal. 2004;24:17-8.

[62] Zhao X Tian B Hao Y Zhang X He Y Li L Survey of blindness and low vision in the middle-aged and elder population in community. [In Chinese]. Chinese Ophthalmic Research. 2009;27:1126-31.

[63] Xie T Chen X Eubulihasumu M Song Y Wang Y Survey of blindness and low vision among Uigur peasants above 40 in Kuche country of Xinjiang. [In Chinese]. Chinese Ophthalmic Research. 2007;25:785-8.

[64] Zhang T Jiang Y Tang J Fan Y An epidemiological survey and the treatment on blindness and low vision among adults aged 45 years or above in Shawan county of Xinjiang, China. [In Chinese]. Chinese Journal of Practical Ophthalmology. 2004;22:934-6.

[65] Zhou J Yuan Y Zhang X Guan HJ A prevalence investigation of blindness and low vision in 2008 among adults aged 60 years or above in 2 villages of Nantong. [In Chinese]. Zhonghua Yan Ke Za Zhi. 2012;48:908-14. 23302246

[66] Tong XW A prevalence investigation of blindess and vision impairment in 2009 in older adults of Dachang Blocks of Baoshan Distict, Shanghai, China. [In Chinese]. Zhonghua Yan Ke Za Zhi. 2011;47:785-90. 22177122

[67] Tang Y Wang X Wang J Huang W Gao Y Luo Y Prevalence and Causes of Visual Impairment in a Chinese Adult Population: The Taizhou Eye Study. Ophthalmology. 2015;122:1480-8. 10.1016/j.ophtha.2015.03.022 25986897

[68] Li J Zhong H Cai N Luo T Li J Su X The prevalence and causes of visual impairment in an elderly Chinese Bai ethnic rural population: the Yunnan minority eye study. Invest Ophthalmol Vis Sci. 2012;53:4498-504. 10.1167/iovs.12-9429 22678502

[69] Li X Zhou Q Sun L Wang Z Han S Wu S Prevalence of blindness and low vision in a rural population in northern China: preliminary results from a population-based survey. Ophthalmic Epidemiol. 2012;19:272-7. 10.3109/09286586.2012.700081 22917525

[70] Yang M Epidemiological survey of visual impairment in Funing County, Jiangsu. [In Chinese]. Zhonghua Yan Ke Za Zhi. 2017;53:502-8. 28728283 10.3760/cma.j.issn.0412-4081.2017.07.006

[71] Yi M. Prevalence of blindness and moderate and severe visual impairment among adults aged 50 years or above in the Shunqing district of Nanchong [In Chinese] [Master Degree]: North Sichuan Medical College; 2015.

[72] Hu J Ji H Wang X Guan H Zhang J Xu Y Cross-sectional study of blindness and low vision among adults aged 50 years or above in Nantong rural area. [In Chinese]. Medical Journal of Communications. 2010;24:133-6.

[73] Zhou W Liu C Wu Z Zhang X Hao Z Li S The investigation of blindness and moderate and severe visual impairment among the elderly aged 60 or above in the southern part of Pudong District. [In Chinese]. Shanghai Journal of Preventive Medicine. 2017;29:78-80.

[74] Wang LH Prevalence of visual impairment and blindness in older adults in rural Shandong Province. [In Chinese]. Zhonghua Yan Ke Za Zhi. 2012;48:226-33. 22800420

[75] Huang S Zheng Y Foster PJ Huang W He M Prevalence and causes of visual impairment in Chinese adults in urban southern China. Arch Ophthalmol. 2009;127:1362-7. 10.1001/archophthalmol.2009.138 19822854

[76] Hou B De J Wu H Gesang D Bu P Qiangba S Prevalence of blindness among adults aged 40 years or above in Linzhou county of Lasa. [In Chinese]. Zhonghua Yan Ke Za Zhi. 2002;38:589-93. 12487906

[77] Wang W A prevalence investigation of blindness and vision impairment in aged people in Langxia Town, Jinshan District, Shanghai, China. [In Chinese]. Recent Advances in Ophthalmology. 2011;31:969-75.

[78] Liu C, Zhou W, Zhao R, Lu Y, Zhang X, Ji P, et al. Analyzing Visual Acuity Status of 70 Years Old and Older People in Pudong New Area. [In Chinese]. Chinese Primary Health Care. 2013;10:85-7.

[79] Deng L. Investigation of the prevalence and the risk factors of blindness and vision impairment with moderate and severe degree among adults aged 50 years and above in Yugan country [In Chinese] [Master Degree]. Nanchang University; 2016.

[80] Zhou J Prevalence of Blindness and Severe Vision Loss in Luxi County of Yunnan Province. [In Chinese]. Journal of Kunming Medical University. 2013;34:51-7.

[81] Wu M. Assessment of the prevalence of blindness among people aged 50 and above in Luliang County of Yunnan Province. [In Chinese]. Journal of Traditional Chinese Ophthalmology. 2011;21:106-9.

[82] Wu M Zhu M Epidemiologic study of blindness in population aged 50 and above of Kunming city. [In Chinese]. Chinese Ophthalmic Research. 2008;26:551-3.

[83] Huang XB Zou H Wang N Wang W Fu J Shen B A prevalence survey of blindness and visual impairment in adults aged equal or more than 60 years in Beixinjing blocks of Shanghai, China. [In Chinese]. Zhonghua Yan Ke Za Zhi. 2009;45:786-92. 20137282

[84] Song W Sun X Shao Z Zhou X Kang Y Sui H Prevalence and causes of visual impairment in a rural North-east China adult population: a population-based survey in Bin County, Harbin. Acta Ophthalmol. 2010;88:669-74. 10.1111/j.1755-3768.2009.01768.x 19900201

[85] Xiong X. The prevalence and causes of visual impairment in an elderly Chinese Tujia ethnic rural population: the Chongqing Minority Eye Study [In Chinese] [Master Degree]: Chongqing Medical University; 2018.

[86] Liang YB Friedman DS Wong TY Zhan SY Sun LP Wang JJ Prevalence and causes of low vision and blindness in a rural chinese adult population: the Handan Eye Study. Ophthalmology. 2008;115:1965-72. 10.1016/j.ophtha.2008.05.030 18684506

[87] Cai N Yuan Y Zhao J Zhong H Ellwein LB Chen M Prevalence and causes of blindness and moderate and severe visual impairment among adults aged 50 years or above in Luxi County of Yunnan Province: the China Nine-Province Survey. [In Chinese]. Zhonghua Yan Ke Za Zhi. 2013;49:801-6. 24330929

[88] Liu H Prevalence of blindness and moderate and severe visual impairment among adults aged 50 years or above in the Shunyi District of Beijing: the China Nine-Province Survey. [In Chinese]. Zhonghua Yan Ke Za Zhi. 2012;48:199-204. 22800416

[89] Lu H Prevalence of blindness and moderate and severe visual impairment among adults aged 50 years or above in Qidong City of jiangsu Province: the China Nine-Province Survey. [In Chinese]. Zhonghua Yan Ke Za Zhi. 2012;48:205-10. 22800417

[90] Lü JH Prevalence and causes of blindness and moderate and severe visual impairment among adults aged 50 years or above in Longyao County of Hebei Province: the China Nine-Province Survey. [In Chinese]. Zhonghua Yan Ke Za Zhi. 2013;49:783-8. 24330926

[91] Ma XZ Prevalence and causes of blindness and moderate and severe visual impairment among adults aged 50 years or above in Changji City of XinjiangUygur Autonomous Region: the China Nine-Province Survey. [In Chinese]. Zhonghua Yan Ke Za Zhi. 2013;49:795-800. 24330928

[92] Yi JL Prevalence of blindness and moderate and severe visual impairment among adults aged 50 years or above in Ji’an county of Jiangxi province: the China Nine-Province Survey. [In Chinese]. Zhonghua Yan Ke Za Zhi. 2012;48:524-9. 22943808

[93] Ge J He M Ellwein LB He N Yang M Wang Y Prevalence of blindness and moderate and severe visual impairment among adults aged 50 years or above in Yangxi County of Guangdong Province: the China Nine-Province Survey. [In Chinese]. Chinese Journal of Ophthalmology. 2014;50:167-72. 24841810

[94] Yin ZQ Prevalence and causes of blindness and moderate and severe visual impairment among adults aged 50 years or above in Yongchuan District of Chongqing City: the China Nine-Province Survey. [In Chinese]. Zhonghua Yan Ke Za Zhi. 2013;49:777-82. 24330925

[95] Zhang L Cui H Zhao J Ellwein LB Li Z Li M Prevalence of blindness and moderate and severe visual impairment among adults aged 50 years or above in Shuangcheng City of Heilongjiang Province. [In Chinese]. Zhonghua Yan Ke Za Zhi. 2014:50:173-8. 24841811

[96] Neng L Huizhong C Pinfang G. Investigation on blindness and low vision of the elderly in Shihua area of Shanghai. Chronic Pathematology Journal. 2019;4:489-92.

[97] Mukesh BN Le A Dimitrov PN Ahmed S Taylor HR McCarty CA Development of cataract and associated risk factors: the Visual Impairment Project. Arch Ophthalmol. 2006;124:79-85. 10.1001/archopht.124.1.79 16401788

[98] Mitchell P Cumming RG Attebo K Panchapakesan J Prevalence of cataract in Australia: the Blue Mountains eye study. Ophthalmology. 1997;104:581-8. 10.1016/S0161-6420(97)30266-8 9111249

[99] Age-Related Eye Disease Study Research Group. Risk factors associated with age-related nuclear and cortical cataract: a case-control study in the Age-Related Eye Disease Study, AREDS Report No. 5. Ophthalmology. 2001;108:1400-8. 10.1016/S0161-6420(01)00626-1 11470690 PMC1473213

[100] Yuan F Qian D Huang C Tian M Xiang Y He Z Analysis of awareness of health knowledge among rural residents in Western China. BMC Public Health. 2015;15:55. 10.1186/s12889-015-1393-2 25637079 PMC4320617

[101] Pincus T Formal educational level–a marker for the importance of behavioral variables in the pathogenesis, morbidity, and mortality of most diseases? J Rheumatol. 1988;15:1457-60. 3204595

[102] Pincus T Callahan LF Associations of low formal education level and poor health status: behavioral, in addition to demographic and medical, explanations? J Clin Epidemiol. 1994;47:355-61. 10.1016/0895-4356(94)90156-2 7730860

[103] Bonaccio M Di Castelnuovo A Costanzo S Persichillo M Donati MB de Gaetano G Interaction between education and income on the risk of all-cause mortality: prospective results from the MOLI-SANI study. Int J Public Health. 2016;61:765-76. 10.1007/s00038-016-0822-z 27091201

[104] Lee YH Chiang T Liu CT Residents’ educational attainment and preventive care utilization in China. Int J Health Care Qual Assur. 2018;31:41-51. 10.1108/IJHCQA-01-2017-0001 29504840

[105] Wang W Yan W Fotis K Prasad NM Lansingh VC Taylor HR Cataract Surgical Rate and Socioeconomics: A Global Study. Invest Ophthalmol Vis Sci. 2016;57:5872-81. 10.1167/iovs.16-19894 27802517

[106] Jin G Xiao W Ding X Xu X An L Congdon N Prevalence of and Risk Factors for Diabetic Retinopathy in a Rural Chinese Population: The Yangxi Eye Study. Invest Ophthalmol Vis Sci. 2018;59:5067-73. 10.1167/iovs.18-24280 30357401

