# Supplementary material for: Prevalence of visual impairment among older Chinese population: A systematic review and meta-analysis
Source: J Glob Health. 2021 May 1;11:08004. doi: 10.7189/jogh.11.08004 (PMC8088771; doi:10.7189/jogh.11.08004)
Supplement: Online Supplementary Document [file jogh-11-08004-s001.pdf]

Figure S1, Panels A-H

A

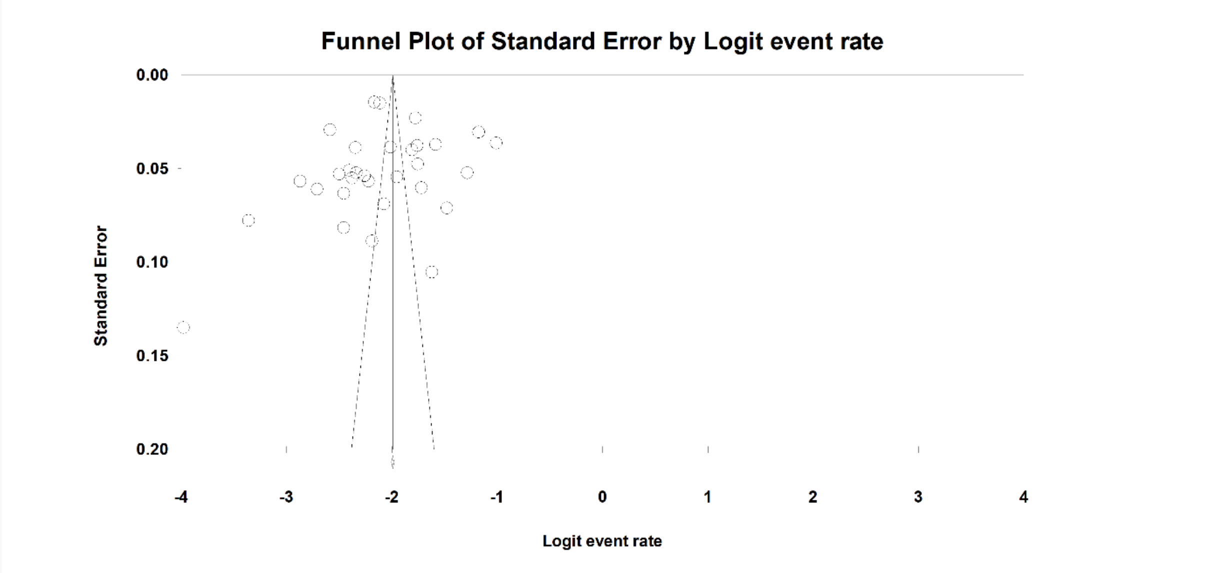

B

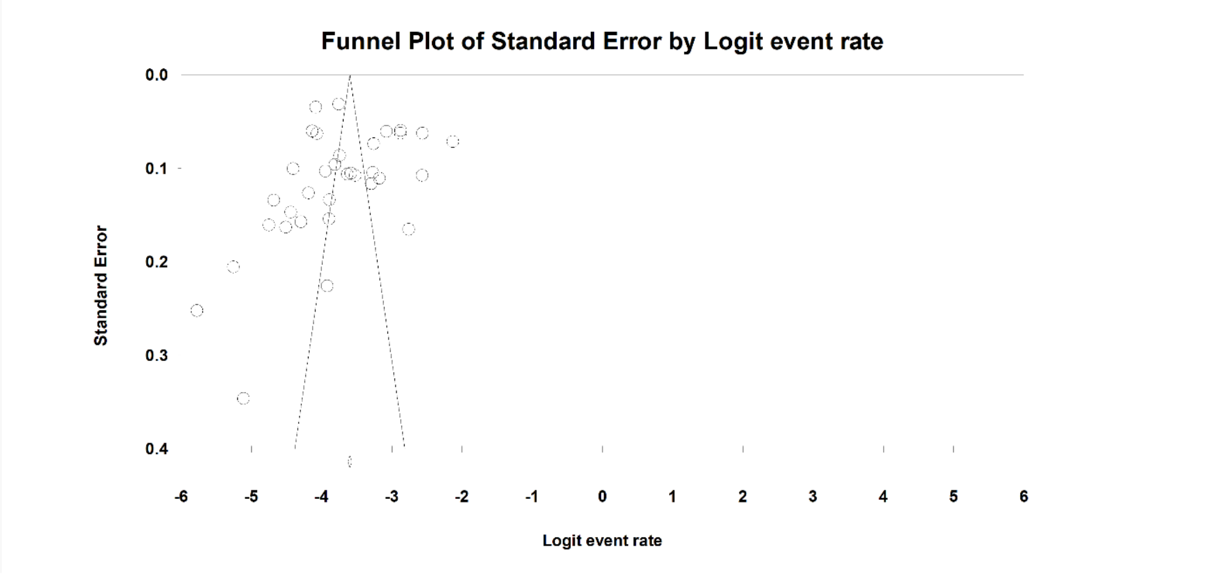

C

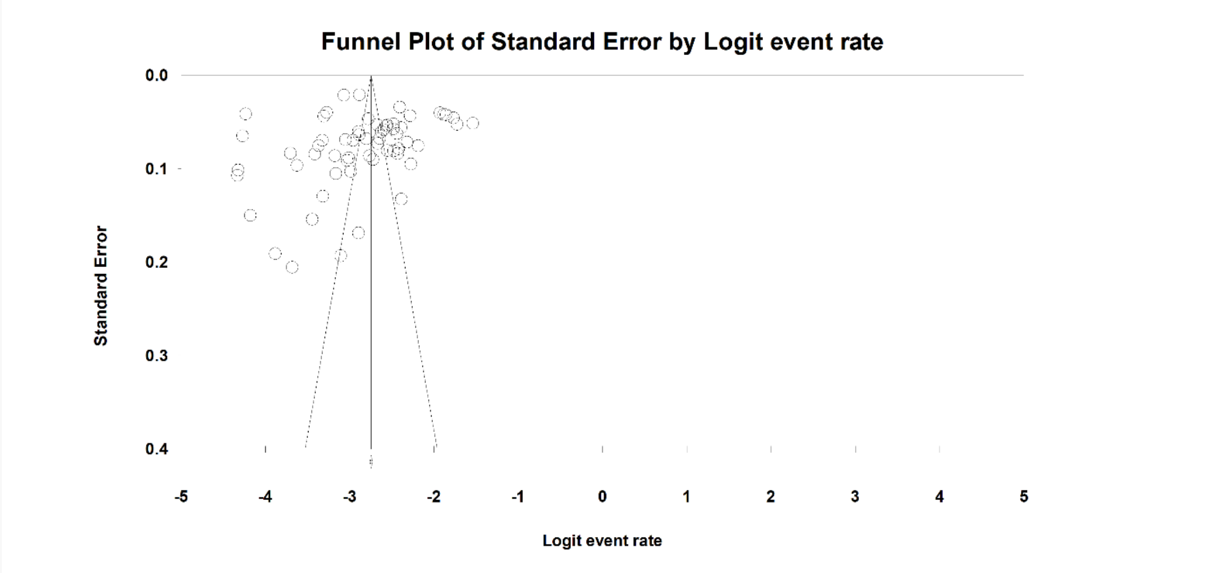
Funnel Plot of Standard Error by Logit event rate
Standard Error
0.0
0.1
0.2
0.3
0.4
-5
-4
-3
-2
-1
0
1
2
3
4
5
Logit event rate

D

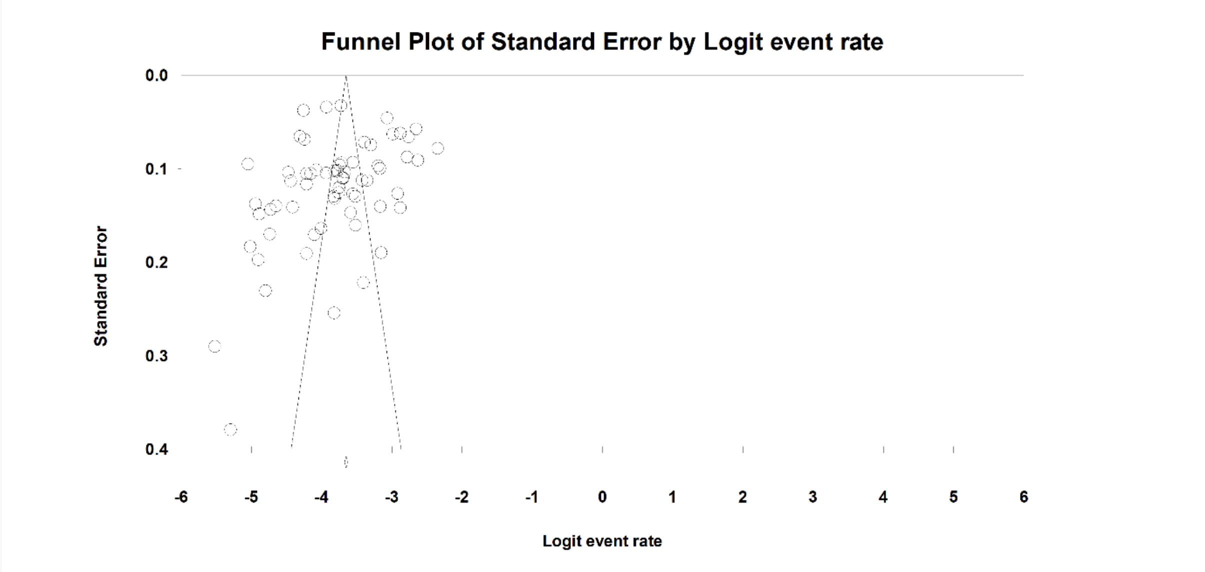
Funnel Plot of Standard Error by Logit event rate
Standard Error
0.0
0.1
0.2
0.3
0.4
-6
-5
-4
-3
-2
-1
0
1
2
3
4
5
6
Logit event rate

E

Study name	Event rate	Lower limit	Upper limit	Z-Value	p-Value
Emmy Y. Li	0.118	0.110	0.126	-52.246	0.000
CW Pan	0.063	0.056	0.070	-44.358	0.000
JF Chen	0.034	0.029	0.039	-43.184	0.000
H Hu	0.147	0.138	0.157	-46.560	0.000
S Liu	0.076	0.069	0.084	-47.176	0.000
L Li	0.018	0.014	0.024	-29.468	0.000
S Wu	0.079	0.071	0.089	-38.788	0.000
ZJ Li	0.083	0.076	0.091	-47.129	0.000
J Zhou	0.186	0.166	0.208	-20.740	0.000
XW Tong	0.088	0.080	0.097	-44.657	0.000
YT Tang	0.088	0.082	0.094	-60.418	0.000
J Li	0.152	0.138	0.168	-28.493	0.000
XF Li	0.095	0.086	0.104	-41.869	0.000
RR Zhu 1	0.236	0.226	0.247	-38.430	0.000
RR Zhu 2	0.054	0.048	0.060	-50.540	0.000
M Yang	0.236	0.226	0.247	-38.442	0.000
MC Yi	0.125	0.113	0.137	-35.773	0.000
JY Hu	0.165	0.139	0.196	-15.367	0.000
W Zhou	0.148	0.136	0.160	-36.775	0.000
LH Wang	0.070	0.066	0.074	-88.099	0.000
W Wang	0.217	0.200	0.235	-24.512	0.000
CJ Liu	0.145	0.140	0.151	-77.108	0.000
LL Deng	0.170	0.160	0.181	-42.587	0.000
JH Zhou	0.141	0.132	0.151	-45.142	0.000
XY Zhou	0.112	0.099	0.126	-30.087	0.000
BL Yuan	0.085	0.077	0.094	-43.157	0.000
SS huang	0.101	0.086	0.118	-24.639	0.000
BJ Hou	0.079	0.068	0.092	-30.069	0.000
XB Huang	0.268	0.254	0.282	-27.671	0.000
WL Song	0.098	0.089	0.108	-39.184	0.000
JL Zhao 2014	0.103	0.100	0.106	-149.018	0.000
JL Zhao 2006	0.108	0.105	0.111	-140.163	0.000
	0.109	0.094	0.126	-25.336	0.000

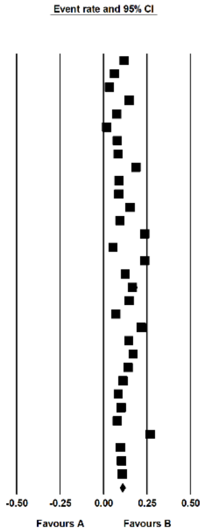

Meta Analysis

F

Study name	Event rate	Lower limit	Upper limit	Z-Value	p-Value
Emmy Y. Li	0.044	0.039	0.049	-50.850	0.000
CW Pan	0.005	0.003	0.008	-25.570	0.000
JF Chen	0.003	0.002	0.005	-22.897	0.000
H Hu	0.054	0.048	0.060	-48.347	0.000
ZQ Li	0.020	0.013	0.030	-17.352	0.000
S Liu	0.053	0.048	0.060	-46.067	0.000
L Li	0.014	0.010	0.018	-27.306	0.000
S Wu	0.027	0.022	0.033	-34.042	0.000
ZJ Li	0.019	0.016	0.023	-38.292	0.000
J Zhou	0.071	0.059	0.087	-23.861	0.000
XW Tong	0.009	0.006	0.012	-29.552	0.000
YT Tang	0.012	0.010	0.015	-43.895	0.000
J Li	0.036	0.029	0.045	-28.272	0.000
XF Li	0.012	0.009	0.016	-30.217	0.000
RR Zhu 1	0.023	0.020	0.027	-43.419	0.000
RR Zhu 2	0.009	0.007	0.012	-34.910	0.000
M Yang	0.023	0.020	0.027	-43.419	0.000
MC Yi	0.029	0.023	0.035	-32.686	0.000
JY Hu	0.060	0.044	0.081	-16.691	0.000
W Zhou	0.011	0.008	0.015	-27.680	0.000
LH Wang	0.016	0.014	0.018	-68.773	0.000
W Wang	0.020	0.015	0.027	-25.264	0.000
CJ Liu	0.017	0.015	0.019	-64.654	0.000
LL Deng	0.022	0.018	0.026	-39.702	0.000
JH Zhou	0.037	0.032	0.043	-44.173	0.000
M Wu1	0.020	0.016	0.026	-29.079	0.000
XY Zhou	0.040	0.033	0.049	-28.699	0.000
BL Yuan	0.015	0.012	0.019	-33.125	0.000
SS huang	0.006	0.003	0.012	-14.760	0.000
BJ Hou	0.106	0.094	0.120	-29.785	0.000
M Wu	0.037	0.030	0.045	-31.255	0.000
XB Huang	0.072	0.064	0.080	-40.999	0.000
WL Song	0.026	0.021	0.032	-34.180	0.000
JL Zhao 2014	0.017	0.016	0.018	-118.128	0.000
JL Zhao 2006	0.023	0.022	0.024	-120.088	0.000
	0.022	0.018	0.028	-34.584	0.000

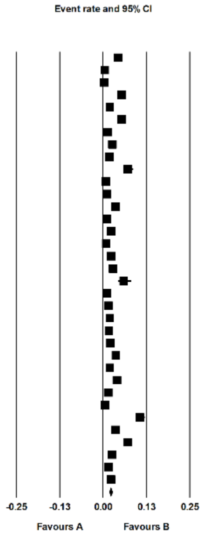

Meta Analysis

G

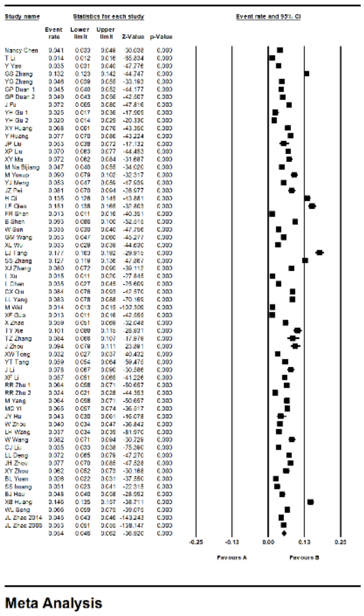

H

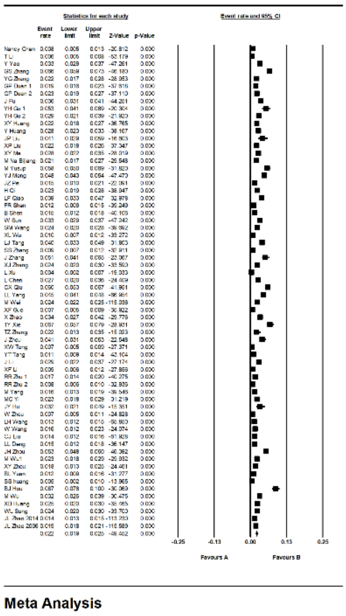

Supplementary figure 1. Funnel plot for prevalence of MSVI by PVA (A); Funnel plot for prevalence of blindness by PVA (B); Funnel plot for prevalence of MSVI by BCVA (C); Funnel plot for prevalence of blindness by BCVA (D); Forest plot for prevalence of MSVI by PVA (E); Forest plot for prevalence of blindness by PVA (F); Forest plot for prevalence of MSVI by BCVA (G); Forest plot for prevalence of blindness by BCVA (H).
